# Dinner to Dr. Balmanno, of Glasgow

**Published:** 1836-01

**Authors:** 


### DINNER TO DR. BALMANNO, OF GLASGOW.

A public dinner was given to Dr. Balmanno on the 18th of November, on the occasion of his retirement from the office of Senior Physician to the Glasgow Royal Infirmary. The chair was taken by Dr. Cumin, who so well expressed, when proposing the health of Dr. Balmanno, every thing appropriate to so interesting an occasion, that we should most gladly extract his remarks from the report, did our limits permit. We read the testimony thus conveyed to a very meritorious officer of a public institution with the more gratification, on account of the too numerous instances which have even recently come to our knowledge in which the officers of such institutions, and candidates for medical appointments in them, have been treated with injustice, and even with indignity. It is, however, we must observe, the younger men alone who submit to these degradations. The older physicians and surgeons shew more self-respect. Dr. Balmanno at least has retired with that which should accompany physicians advanced in years and experience; and the honours paid to him on his retirement bear testimony to the honourable public career he has thus finished.

